# Nutrigenomics as a tool to study the impact of diet on aging and age-related diseases: the *Drosophila* approach

**DOI:** 10.1186/s12263-019-0638-6

**Published:** 2019-05-02

**Authors:** Zoi Evangelakou, Maria Manola, Sentiljana Gumeni, Ioannis P. Trougakos

**Affiliations:** Department of Cell Biology and Biophysics, Faculty of Biology, National and Kapodistrian University of Athens, 15784 Athens, Greece

**Keywords:** Aging, Diet, *Drosophila*, Foxo, Insulin signaling, Nrf2, Nutrigenomics, Nutrition, Tor

## Abstract

Aging is a complex phenomenon caused by the time-dependent loss of cellular homeodynamics and consequently of physiological organismal functions. This process is affected by both genetic and environmental (e.g., diet) factors, as well as by their constant interaction. Consistently, deregulation of nutrient sensing and signaling pathways is considered a hallmark of aging. Nutrigenomics is an emerging scientific discipline that studies changes induced by diet on the genome and thus it considers the intersection of three topics, namely health, diet, and genomics. Model organisms, such as the fruit fly *Drosophila melanogaster*, have been successfully used for in vivo modeling of higher metazoans aging and for nutrigenomic studies. *Drosophila* is a well-studied organism with sophisticated genetics and a fully annotated sequenced genome, in which ~ 75% of human disease-related genes have functional orthologs. Also, flies have organs/tissues that perform the equivalent functions of most mammalian organs, while discrete clusters of cells maintain insect carbohydrate homeostasis in a way similar to pancreatic cells. Herein, we discuss the mechanistic connections between nutrition and aging in *Drosophila*, and how this model organism can be used to study the effect of different diets (including natural products and/or their derivatives) on higher metazoans longevity.

**Electronic supplementary material:**

The online version of this article (10.1186/s12263-019-0638-6) contains supplementary material, which is available to authorized users.

## Introduction

*Drosophila melanogaster* has been used for long as a vanguard model organism for genetic studies and for the analysis of molecular mechanisms underlying development, behavior, and diseases. Also, its unique features make *Drosophila* an effective experimental model for aging research as it has a relatively small body size; a very rapid life cycle (~ 10–14 days depending on the environmental temperature) and a quite short lifespan, which is inversely proportional to increased temperature and fecundity [1]. Furthermore, *Drosophila* has four different developmental stages, namely, the embryo, larva, pupa, and adult. Since each developmental stage has its own specific experimental advantages, the fly may be considered as a model of multiple organisms that can be dissected and genetically manipulated [2]. Moreover, *Drosophila* is comparatively easier and cheaper (as compared, for instance, to mice) to maintain in large numbers and has a relatively low cost of rearing and housing. Given the genetic tractability and the many tools available for forward and reverse genetics (e.g., the GAL4/UAS system, RNAi, CRISPR/Cas9, transposon-mediated mutagenesis or excision, chemically induced mutations, etc.), studies can be performed more rapidly, including those that refer to the development of human disease models [3–6].

**Fig. 1:**
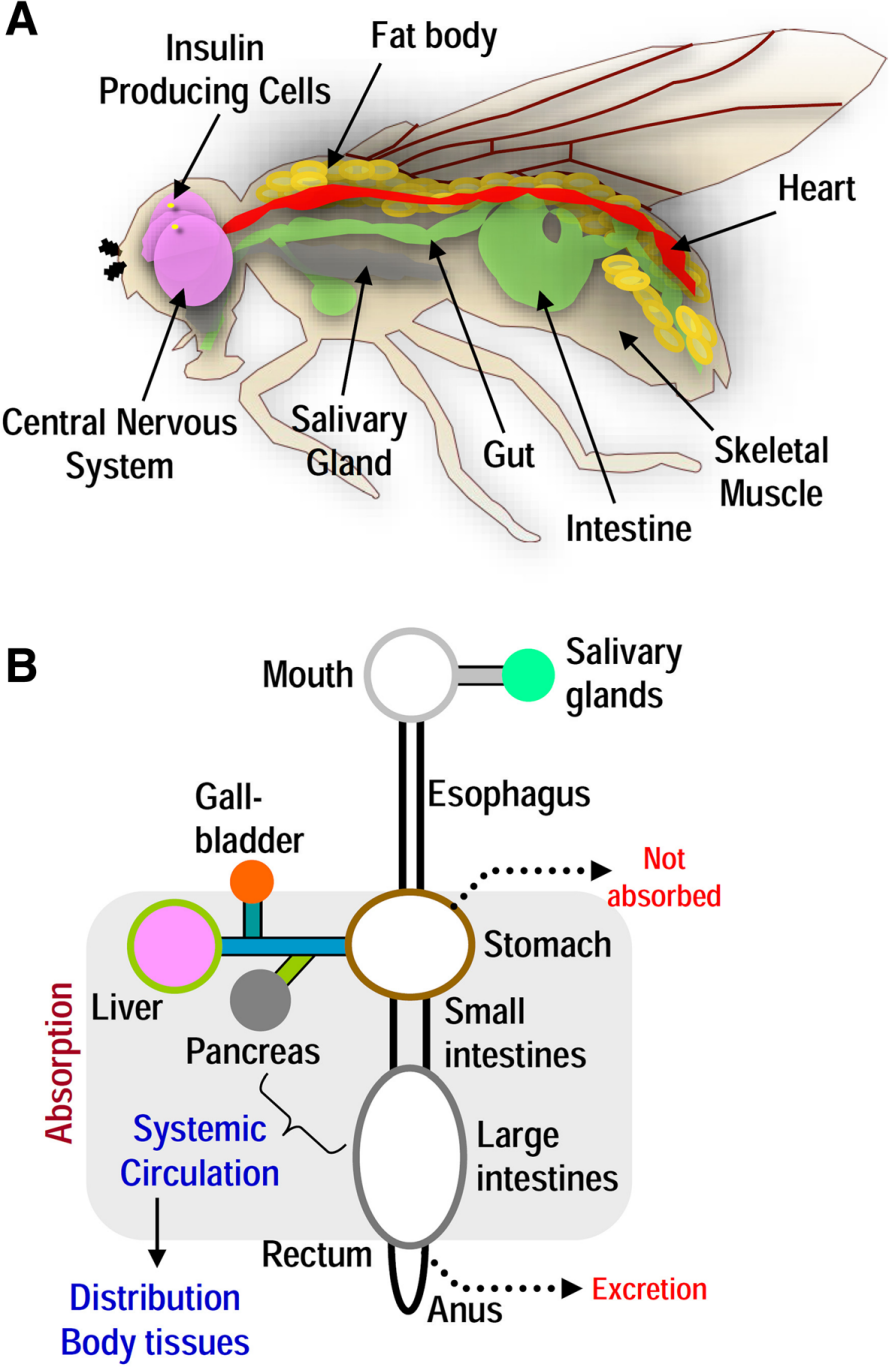
*Drosophila melanogaster* as a model organism for nutrigenomics and its translational impact. **a** The fruit fly has emerged as an excellent model organism to study nutrigenomics in aging and age-related diseases. *Drosophila* is well-suited in this line of research due to the highly annotated and significantly conserved (compared to mammals) genome. Notably, ~ 75% of disease-related genes in humans have functional orthologs in the fly, while there are significant similarities in organs that perform the equivalent functions of the mammalian heart, lung, kidney, gut, liver, adipose tissue, and reproductive tract. *Drosophila* is characterized by well-developed and complex neural and circulatory systems; the latter is composed of a pumping heart tube that through hemolymph circulates regulatory molecules (e.g., insulin-like peptides) to peripheral tissues. Discrete clusters of cells in the brain, muscle, and fat body maintain insect carbohydrate homeostasis in a way similar to pancreatic α- and β-cells. *Drosophila* exerts several complex physiological functions, such as nutrients digestion, absorption, and post-absorption processes making this organism an ideal in vivo experimental platform for nutrigenomics studies. **b** As most of the components of the human digestive system (shown here diagrammatically) have equivalent modules in the fly model, the latter can be used in nutritional sciences and nutrigenomics

The fly genome is completely sequenced and encodes ~ 14,000 genes, of which more than 60% share homology with human genes. Moreover, approximately 75% of disease-related genes in humans have a functional homolog in the fly and many of the physiological pathways, such as superoxide metabolism, insulin-like signaling, DNA damage and antioxidant responses, proteostatic, and mitostatic networks, are highly conserved between *Drosophila melanogaster* and vertebrates [7–10]. *Drosophila* have organs/tissues that are equivalent to mammalian nervous system, heart, digestive system, kidney, adipose tissue, and reproductive tract [11–13] (Fig. 1); also, flies display complex behaviors and responses such as active and rest periods, mating, responses to alterations in temperature and food composition, and also a complex circadian cycle [14, 15].

**Fig. 2:**
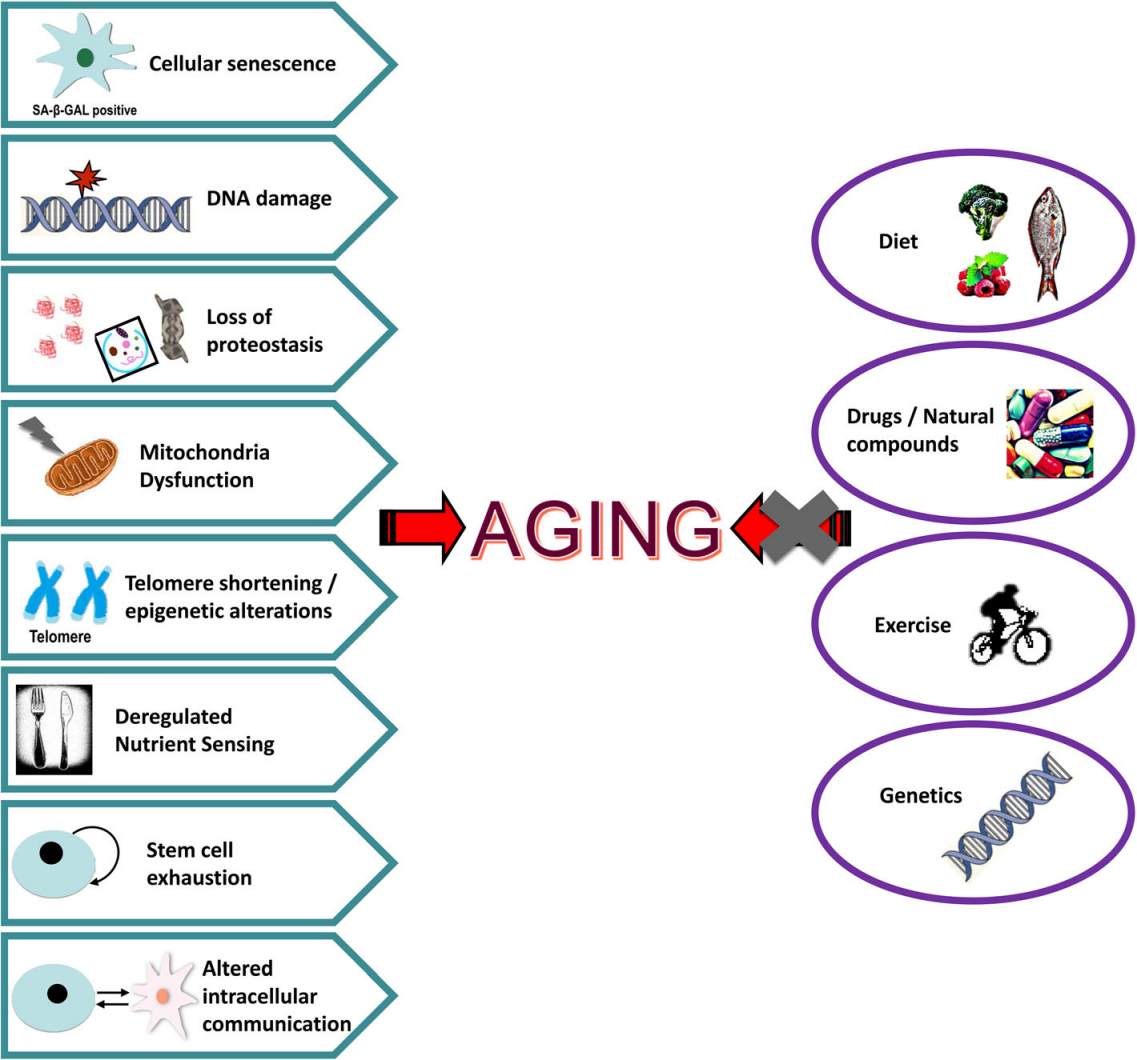
The hallmarks of aging. Aging hallmarks are common molecular processes and phenotypic alterations that define cellular senescence and/or systemic aging across evolution. The highly conserved aging traits may act independently or coordinately with exogenous or endogenous stress factors, including specific dietary habits. Given that neither genetic modifications nor caloric restriction can be applied in humans; the understanding of how nutrition alters genome (nutrigenomics) and consequently proteome expression patterns, is a critical parameter for the design of nutritional interventions aiming to increase healthy aging

Aging is a complex stochastic process of progressive accumulation of biomolecular damage that varies between individuals due to the interplay of genetic and environmental factors. Consequently, aging is invariably characterized by several distinct signs known as hallmarks of aging (Fig. 2). These include genomic instability, telomere attrition, epigenetic alterations, loss of proteostasis, deregulation of nutrient sensing/signaling, mitochondrial dysfunction, cellular senescence, stem cell exhaustion, and alteration of intercellular communication [16, 17]. These hallmarks lead to a progressive loss of organismal integrity and homeodynamics, which eventually results in impaired cellular function and increased morbidity. As in all other metazoans, aging in *Drosophila* correlates with increased mortality rates, and it is also marked by decreased spontaneous movement and climbing speed, impaired memory, heart function, and reproductive capacity [18–21].

Nutrigenomics is a rapidly emerging research field that studies the changes induced on the genome by diet, and it thus considers the intersection of three topics, namely, health, diet, and genomics. Nutrigenomics can be mainly conducted through the various *–omics* techniques, which (among others) include microarrays or RNA-Seq analysis (transcriptomics) for the measurement of changes in mRNAs expression; proteomics that identify changes in polypeptides expression or in post-translational modifications; metabolomics that mainly focus on the study of metabolites with molecular weight less than 2000 Da, and also, epigenomics that measure the changes in the epigenome, i.e., the histone post-translational modifications and/or the DNA methylation pattern. Given its versatility as an experimental model, *Drosophila* is widely used for *–omics* analyses, and it can therefore be used to conduct many types of nutrigenomic studies [22]. At a more advanced stage, nutrigenomic studies and the understanding of diet-disease relationships can be used for the development of personalized dietary and medicinal products.

Herein, we discuss the mechanistic connections between nutrition and aging in *Drosophila*, and how this model organism can be used (with possible limitations) to study the effect of different diets (including natural products and/or their derivatives) on higher metazoans longevity. Also, we summarize the nutritional interventions that promote healthy aging and/or longevity in flies.

## Molecular links between nutrition and aging in *Drosophila*

**Fig. 3:**
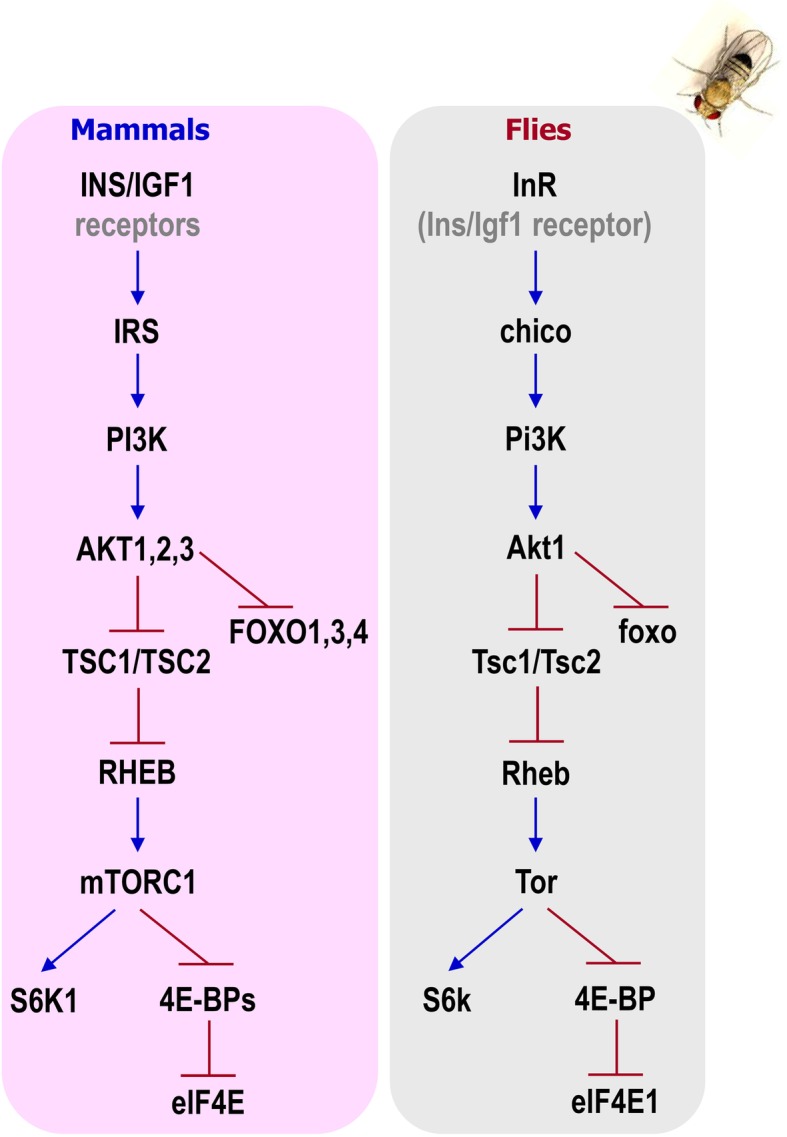
Evolutionary conservation of the IIS pathway. Comparative depiction of the IIS pathway regulatory components and their evolutionary conservation in mammals and in *Drosophila*

Deregulation of the cellular metabolic pathways and nutrient sensing is a major molecular modification that drives age-related damage responses from yeast to primates [11, 16]. Organisms have developed numerous signaling pathways for nutrient sensing controlled by a highly regulated neuroendocrine system and characterized by excessive interorgan communication, in order to monitor nutrient availability and adjust their “real-time” nutritional status [23, 24]. Moreover, many research groups have highlighted the role of caloric intake or dietary supplementation in lifespan elongation at different model organisms [25–28]. Notably, insulin/insulin-like growth factor signaling (IIS) modules are significantly conserved among mammals and *Drosophila* (Fig. 3).

Towards the trend of direct gene-disease association, several genes of the *Drosophila* genome have been associated with age-related phenotypes arising from nutrient sensing or signaling deregulation. The vast majority of these genes are implicated in a wide range of cellular processes including cell growth and maintenance, metabolism, signal transmission, protein transport, cell communication, stress responses, responses to pathogens, immune responses, oogenesis, and fecundity [29–31]. Typical examples of nutrients sensing/signaling genes that are implicated in *Drosophila* aging are Sirtuin 1 (*Sirt1*, also known as *Sir2*) [32], Insulin-like receptor (*InR*) [33–35], the insulin-like receptor substrate (*chico*) [36, 37], and the forkhead box, sub-group O (*foxo*) gene [38–40] (for a list of cited genes see, Additional file 1: Table S1).

Moreover, genetic manipulations of genes implicated in stress responses, regulation of proteome homeodynamics or energetic pathways and mitochondrial biogenesis, such as the heat shock proteins family (*Hsps*) [41, 42], the transcription factor cap-n-collar isoform-C (*cncC*, the ortholog of the mammalian nuclear factor, erythroid 2 like 2; *Nrf2*) [43, 44], the regulatory particle non-ATPase 11 (*Rpn11*) [45, 46], the autophagy-related 8a (*Atg8a*) [47, 48] and spargel (*srl*, the homolog of the mammalian peroxisome proliferator-activated receptor (PPAR)γ coactivator-1; *PGC-1*) [49, 50] have revealed the functional involvement of these genes in regulating aging progression. Moreover, upon dietary manipulations, another fly gene suggested to influence several longevity traits is the stress-responsive gene methuselah (*mth*) [51, 52].

In line with these findings, molecular nutrient sensors like the AMP-activated protein kinase α subunit (AMPKα) or sirtuins that encode a conserved family of nicotinamide adenine dinucleotide (NAD^+^)-dependent protein deacetylases; sense alternations in cellular energetics as they are affected by either the ratio of ADP/AMP to ATP or NAD^+^ to NADH levels respectively, and therefore accordingly regulate catabolic and anabolic processes [53, 54]. The relative abundance of these cofactor pairs ensures metabolic homeostasis through the transcription of their downstream targets, which eventually modulate longevity [11]. In *Drosophila*, there are two major and highly conserved nutrient signaling pathways, namely, the IIS and the target of rapamycin (Tor) pathways [55], which are sensitive to changes in the cellular levels of glucose and amino acids respectively [56], and coordinately regulate each other [57]. Recent studies have shown that the function of this complex nutrient-sensing mechanism is (directly or indirectly) dependent on different types of diet and nutrients. More specifically, certain dietary interventions that lower the intensity of the signal by targeting modules of these two pathways could result in lifespan extension, improved neuromuscular activity, and preservation of cardiac health during aging [58].

Regardless of the thorough study of aging pathways associated with nutrition, the exact mechanism by which dietary interventions modulate longevity remains elusive. Most probably the coordinated action of a cluster of genes involved in stress responses to oxidants, IIS pathway, apoptosis, programmed autophagy, and the olfactory system, are responsible for the benefits of reduced nutritional input on healthspan and/or lifespan extension [59].

### The IIS pathway

Association studies have shown that the main longevity-related genes involved in nutrient signaling are functionally conserved between the human and the *Drosophila* genome (Fig. 3) [60]. In mammals, energy homeostasis is tightly regulated by the antagonistic action of glucagon and the IIS pathway, as the main circulating energy sources are sugars. In flies, although glucose can be found in the hemolymph, trehalose (Treh) is the prevailing circulating sugar [61], which due to its chemical properties can transiently accumulate in the circulation at high levels without significant detrimental effects; unlike glucose in mammals that leads to hyperglycemia [62]. Nevertheless, overaccumulation or shortage of trehalose (*Treh* null mutants) may diminish the adaptation rates in nutrients poor environments [63].

As mentioned, the IIS pathway is highly conserved in the fruit fly, and *Drosophila* genome encodes eight insulin-like peptides (Ilps 1 to 8) with pleiotropic functions. Ilps are produced in distinct cell and tissue types at different developmental stages and bind to a single InR [64, 65]. Ilp2, Ilp3, and Ilp5 are produced and secreted by insulin-producing cells (IPCs). IPCs are functional homologs of the human β-pancreatic cells and are located in the median neurosecretory cluster of flies’ brain [64]. Upon secretion of the Ilps in the circulatory system (i.e. the hemolymph), a cascade of signal transduction (that employs several kinases), results in the suppression of the longevity-associated transcription factor foxo [66] (Fig. 3). The IIS pathway in *Drosophila* positively regulates fat storage and glycogen synthesis [63]. Upon low sugar levels in the hemolymph, the α-pancreatic-like cells of the endocrine organ called *corpora cardiaca* activate the internal AMPKα, which triggers the release of the glucagon-like adipokinetic hormone (Akh); a regulator of glycemia and lipid catabolism [61, 62, 67]. Akh binds to the adipocinetic hormone receptor (AkhR) in target tissues and triggers the conversion of stored glycogen and lipids to free energy [68]. Reduced activity of the IIS pathway is also associated with reduced growth and limited reproduction rates [36, 69]. The fat body (analog of the mammalian liver and adipose tissue) is the main nutrient sensing organ, which remotely regulates the secretion of Ilps and longevity [24, 38, 39]. Genetic manipulations which suppress the IIS pathway, such as deletion of the *Ilp2*, *3* and *5* genes; overexpression of *Ilp6* in the fat body or removal of neurosecretory cells from the *Drosophila* brain (IPCs ablation), have revealed the prevailing role of Ilps and *Drosophila* fat body as sensors of nutritional alternations [69–72]. Interestingly, the effects of IIS on longevity are apparently related to both metabolic and proliferative homeostasis since mild suppression of the IIS pathway in certain tissues or cells, which culminates in tissue/cell-specific *foxo* activation, or genetic induction of tissue/cell-specific *foxo* overexpression, result in increased longevity [73]. Moreover, in response to dietary sugars and fats unpaired 2 (upd2), the functional homolog to the mammalian leptin, is produced from the *Drosophila* fat body, which in turn increases Ilps release from IPCs [24]. Notably, the induction of the IIS pathway results in the activation of the major oxidant/electrophile sensitive transcription factor cncC/Nrf2, which triggers the transcriptional activation of antioxidant, proteostatic, and/or mitostatic genes [43, 74, 75]; consistently, cncC/Nrf2 has also been proven to have a regulatory role in energy metabolism [75, 76]. As the cncC/Nrf2 pathway is affected by the nutritional status, certain dietary interventions have the potential to modulate organisms’ detoxification mechanisms, and therefore delay either the onset of age-related diseases or in vivo aging [77, 78].

### The Tor signaling pathway

Sugars mainly serve as the cells’ energy currency, while amino acids mostly serve as building blocks for protein synthesis. As mentioned above, the extra- or intracellular levels of amino acids are sensed by the Tor signaling pathway [79], which plays a vital role in balancing anabolic/catabolic rates, regulating cell growth and affecting longevity [80]. Tor signaling is conserved across evolution, while genetic studies have revealed that inhibition of Tor through nutrition ensures proteostasis and promotes longevity in *Drosophila* by suppressing the IIS pathway and increasing autophagic rates [81–83].

Central to the Tor signaling pathway is the Tor kinase that in mammals joins two multi-protein complexes, namely, the target of rapamycin complex 1 and 2 (TORC1, TORC2) [84]. TORC1 regulates mRNA translation and cell growth by two downstream molecules, namely, the ribosomal protein S6 kinase (S6k) and the cap-dependent translation initiator Thor (or 4E-BP) [85, 86]. TORC2 is involved in actin organization and upon activation, it triggers the phosphorylation of AKT serine/threonine protein 1 (Akt1), the core kinase of the IIS pathway [82]. Several studies point out that the cross-wiring between the two Tor-regulated signal transduction cascades is rather complex since the outcome of any intervention strongly depends on the intensity and the duration of the signal and/or the cell or tissue type [81, 87]. Positive upstream Tor regulators are major modules of the IIS pathway, such as growth factors, Pi3K21B (PI3K), and Akt1 [79], whereas the master nutrient sensors AMPKα and Sir2 negatively regulate Tor activity [88]. Upon nutrient sensing in the *Drosophila* fat body, Tor generates a humoral signal that modulates IIS and growth in peripheral tissues [56], suggesting that the two nutrient signaling pathways do not act independently but there is rather a coordinated action and eventually crosstalking.

## The effects of distinct nutritional interventions on healthy aging

As the prevalence of obesity along with malnutrition rises worldwide, the interest of the scientific community has shifted towards the expansion of nutritional sciences and nutrigenomics [22, 89]. The major aim across these lines of research is to fully address the mechanistic insights of the role of nutrition and nutrient-sensing pathways in promoting healthspan. Genome-wide association studies from human and animal models, the ongoing establishment of molecular mechanisms underlying diseases and the development of advanced analytical techniques for bioevaluation processes, point out the potential benefits of dietary manipulations as a novel anti-aging and/or disease-preventing strategy [2, 57, 89–91]. For example, the use of nutrient-dense foods improves the nutritional status and late-life disabilities of the elderly, the intermittent fasting lowers blood glucose in obese subjects, while caloric restriction extends lifespan and reduces genomic instability of some animal models, serving also as a potential anticancer approach with minimal side effects [92].

### Caloric restriction

Up to date, the most effective and reproducible dietary intervention known to extend lifespan in several animal models including primates is caloric restriction (CR). CR refers to ~ 20–40% reduction of food consumption [93, 94]. About half a century ago, it was reported for the first time in *Drosophila* that diluted medium prolongs both median and maximum lifespan [95, 96]. Like all living organisms, *Drosophila* needs to deploy macro- and micro-nutrients from its environment in order to maintain vital functions such as reproduction, movement, and self-preservation [97]. Although it is known that the nutrients which mostly affect longevity are carbohydrates, proteins, lipids, vitamins, and minerals, their exact mode of action is not well understood [98]; yet, studies in flies suggested that by restricting all dietary components or by simply reducing flies’ protein intake, longevity can be extended by almost 50% [96, 99].

Accordingly, further studies revealed the lifelong beneficial effects of feeding on specific nutrients such as low casein or low intermediate levels of methionine [100, 101], while other studies unveiled the lifespan-shortening effects of feeding on increased essential amino acids without the supplementation of carbohydrates, lipids, or vitamins, pointing out the negative impact of overconsumption [102]. Moreover, restriction of dietary protein suppresses the pathophysiological effects of in vivo organismal aging, reduces disease-associated risk factors, and delays the onset or progression of age-associated diseases [73]. Overall, reducing protein in relation to carbohydrate intake seems to be the key to longevity [103]; this fact contrasts previous studies which promoted CR as the key to enhanced longevity. Specifically, although CR indeed slows down biological aging [104], according to the CALERIE (Comprehensive Assessment of Long-Term Effects of Reducing Intake of Energy Clinical Trials; Gov. Identifier: NCT00427193, 93) clinical trial, it is difficult to enforce long-term CR on humans without detrimental effects on the quality of living [105]. Therefore, scientists have switched to pursuing either periodic dietary restriction (DR) or using small molecules that act as CR mimetics (CRMs), i.e., molecules which reproduce the systemic effects of chronic CR without limiting the amount of food [106, 107].

### Dietary restriction and caloric restriction mimetics

In *Drosophila* rapid (~ 48 h) DR alters the expression of several genes of the IIS/Tor pathways in order to achieve dietary balance [27, 108]. However, the exact molecular mechanism by which low-protein intake leads to lifespan extension needs further investigation, as genetic models prove that it engages both IIS-dependent and IIS-independent mechanisms [109]. In support, long-lived *chico* mutants did not respond to optimal DR suggesting that lifespan extension is based on the IIS pathway [110], whereas *foxo* mutant flies were still sensitive to DR suggesting an IIS independent pathway [109].

On the other hand, *Drosophila* has also been employed as a platform to track molecules that could potentially mimic the beneficial effects of chronic CR, namely, CRMs. The best-studied types of CRMs in the fly model are those which act on specific downstream modules of the nutrients sensing or signaling pathways [107]. However, the use of CRMs does not always result in longevity extension. For instance, metformin, a well-known antidiabetic drug that triggers the activation of the nutrient sensor AMPKα and induces fat burn in the adipose tissue, does not confer lifespan elongation [111]. Administration of the Tor inhibitor rapamycin, known for its immunosuppressant properties, extends in a sex-dependent manner the lifespan of *Drosophila* flies with impaired energy regulation fed on a regular diet [112, 113]. Furthermore, specific concentrations of the food supplement resveratrol promote longevity of flies fed with high lipids by activating the sirtuins network [114]. Another compound recognized as CRM is spermidine, which belongs to the polyamines group; reportedly, spermidine expands the lifespan of healthy *Drosophila* by inducing autophagy [115].

### High-fat and high-sugar diets

**Fig. 4:**
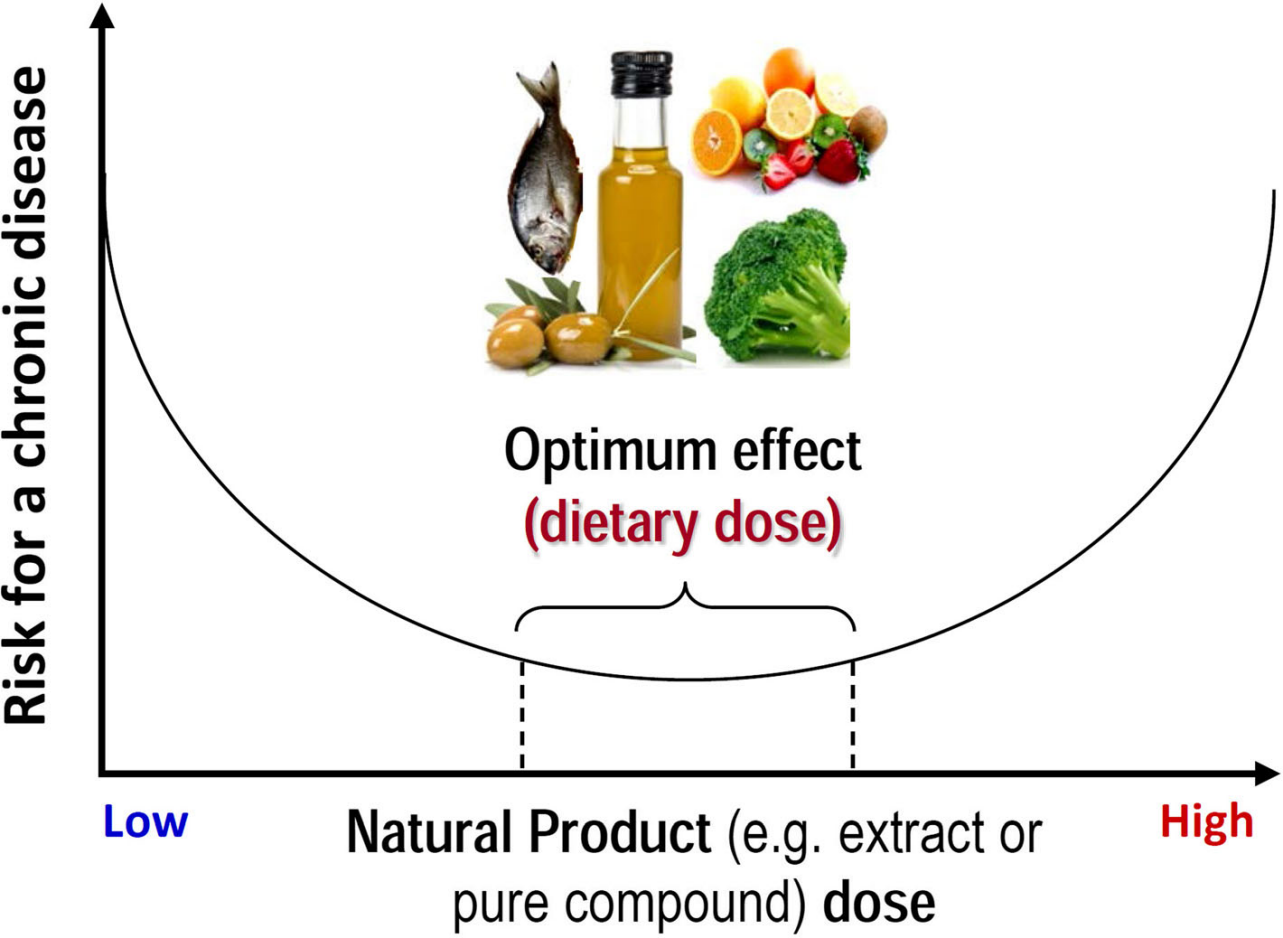
The optimum effect of diet on aging and disease is usually around a narrow dose range. Dietary deficiencies or excess amounts of nutrients can lead to significant adverse effects on healthspan as the dose-response is not linear

*Drosophila* has also served as a model to study complex and progressive metabolic dysregulation during aging. More specifically, high-fat (HFD) and/or sugar (HSD) diets have been used to trigger chronic metabolic diseases like obesity, hyperglycemia, insulin resistance, type II diabetes, and cardiomyopathies [91, 116, 117]. Either plant or animal-derived HFDs increase ectopic fat accumulation, promote insulin resistance, and over-activate the immune system, which in turn shortens lifespan [118–120]. Nevertheless, the effects of HFDs can be partially ameliorated by endurance training [121]. On the other hand, HSDs (containing ~ 30% sugars in the form of sucrose, glucose or fructose) affect Ilps production and lead to peripheral insulin resistance [122, 123]. Along with hyperglycemia and obesity, high-sugar content leads to proteotoxic stress conditions, such as increased endoplasmic reticulum stress, disruption of gut homeostasis, and progressive heart failure [116, 124, 125]. There are several contradictory findings on the effects of HSDs on lifespan, as according to some studies HSDs suppress longevity, whereas others revealed that flies that have overcome HSDs toxicity during development or early adulthood, could extend their lifespan probably through metabolic reprogramming [126–128]. In addition, as reviewed recently [60] and according to the Nutritional Geometric Framework [103], a carbohydrate-rich diet could confer lifespan extension properties if it is accompanied by protein restriction [129]. Overall, these findings highlight that the most compelling aspect for a long healthy living is rather the dietary balance along with specific doses and not actually the caloric reduction (Fig. 4) [130].

## Dietary supplements for healthy aging and as interventions in age-related diseases

Extracts from various sources of the biosphere (e.g., plants, microbes, or marine organisms) have been used for long as food supplements to promote health and/or longevity [131]. Recently, several natural products in the form of extracts or pure compounds have been shown to prolong lifespan and/or lower the risk of age-associated diseases in model organisms by modulating the aforementioned nutrient sensing and signaling pathways. Mechanistically, the modulation of these pathways results in the activation of several cytoprotective processes including autophagy, antioxidant, proteostatic, and DNA repair responses [132–134].

### Plant extracts

Many plant extracts including blueberries, apples, rosemary, ginger, aronia, pomegranate, nectarine, *Rhodiola rosea*, *Platanus orientalis*, asparagus, cocoa, and *Curcuma longa* have been shown to exert beneficial effects in aging studies in *Drosophila*. Blueberries and apples are fruits that possess a great antioxidant capacity due to their polyphenols [135]. Peng and colleagues [136] found that both blueberry (5 mg/ml) and apple polyphenol (10 mg/ml) extracts could significantly extend the mean lifespan of fruit flies by almost 10%. The authors suggest that the lifespan-prolonging effect of these two extracts could be attributed to their interaction with superoxide dismutase (*Sod*) and catalase (*Cat*), which were upregulated, whereas *mth* and *Rpn11*, were found to be downregulated [136, 137]. Similarly, supplementation with a rosemary extract delayed aging in a dose-dependent manner; at 3 mg/ml this extract extended death time by 22.9%, average lifetime by 17.49% and maximum longevity by 12.0%. Moreover, it improved antioxidant enzymes activity, inhibited lipid peroxidation; significantly decreased malondialdehyde (MDA, a lipid oxidation product) content and increased the activities of Cat and Sod [138]. Another extract found to alter the expression patterns of *Cat*, *Sod*, and *mth* was an extract from ginger. Specifically, mRNA expression analysis in 30 days old flies fed with 1 mg/ml of ginger extract showed a significant upregulation of *Sod* and *Cat* genes, whereas downregulation of *mth* was observed in flies fed with 2 mg/ml of the ginger extract as compared to flies fed with a standard diet. Supplementation of the culture medium with 1 mg/ml and 2 mg/ml of ginger extract could prolong the mean lifespan by 6.49% and 7.30%, and the maximum lifespan by 11.97% and 4.66%, respectively. Ginger extract could also regulate the metabolism of amino acids, carbohydrates, and lipids, which indicates that the anti-aging effect is achieved by protecting mitochondrial function, coordinating the oxidant-antioxidant balance and ameliorating metabolic dysfunction [139].

Likewise, 2.5 mg/ml of *Aronia* extract was found to extend the mean lifespan of fruit flies by 18% and significantly improved the locomotor activity of both 10 and 40 days old flies. In 40 days old flies, ROS production was significantly lowered and accumulation of the lipid oxidation product MDA was markedly decreased. The extended longevity and improved locomotion were attributed to increased levels of the antioxidant enzymes Sod, Cat, and glutathione peroxidase (GPx) and to the induction of stress resistance genes, namely, heat shock protein 68 (*Hsp68*), lethal (2) essential for life [*l(2)efl*], and thioredoxin peroxidase 1 (*Jafrac1*) [140]. Furthermore, supplementation with 10% (*v*/*v*) pomegranate juice was shown to extend the lifespan of male flies by 18% and female flies by 8%, when flies were raised separately, whereas a 19% lifespan increase was noted when male and females flies were cultured together. Moreover, researchers observed a simultaneous two-fold enhancement in fecundity and climbing activity, improved resistance to hydrogen peroxide (H_2_O_2_) and paraquat (acute exposure) induced oxidative stress and enhanced resistance to *Candida albicans* infection [141]. Two other studies have identified a *Rhodiola rosea* extract (traditional western Ukraine medicinal adaptogen) as a culture medium additive that could extend lifespan. In the first study, flies fed with 5 mg/ml or 10 mg/ml of a *R. rosea* rhizome powder displayed a 14% to 17% increase in median lifespan; also, flies were physically more active and less sensitive to oxidative and heat stress compared to controls. All the aforementioned effects were more pronounced at middle-aged flies [142]. In addition, Schriner and colleagues [143] found that a *R. rosea* extract extends lifespan in both genders, yet exerting some sex-specific differences. In female flies, the expression levels of glycolytic and *Sir2* genes along with NADH levels were downregulated, while in males the *R. rosea* extract downregulated the mitochondrial heat shock protein 22 (*Hsp22*) expression levels, provided no protection against heat stress and had no effect on heat shock protein 70 (*Hsp70*) gene expression [143]. Furthermore, we recently reported that the extracts of the *Platanus orientalis* activated proteostatic mechanisms, e.g., proteasome and lysosomal cathepsins activity, ameliorated age-related phenotypes, and promoted *Drosophil*a longevity by activating tissues’ antioxidant responses [144].

Likewise, SC100, a preparation which consists of four herbal extracts containing *Astragalus membranaceus* root, *Pterocarpus marsupium* bark, pine bark oligo-proanthocyanidins, and *L-theanine* predicted to modulate the expression of many age- and stress-related genes and it extended the longevity of *Drosophila* flies under certain environmental conditions, such as housing size and population density [145]. Last but not the least, exposure to extracts from two commonly used Indian medicinal plants, namely, *Curcuma longa* (rhizome) and *Emblica officinalis* (fruit) could significantly increase flies’ lifespan [146]. Thus, extracts obtained from plants provide a precious source of natural products that can improve healthspan and/or promote longevity.

### Plant-derived compounds

Resveratrol is a stress-response lipophilic polyphenol produced by plants, which has been shown to extend lifespan in different organisms through its CRM properties. Supplementation of larval diet with resveratrol has been found to extend the longevity of both genders and increase locomotor activity in adult males. This effect was attributed to the increased activity of the Sod and Cat enzymes in both genders [147]. Moreover, resveratrol could extend the lifespan of female flies fed with HFDs. This was associated with the suppression of age-related pathways, by downregulation of antioxidant peroxiredoxins, insulin-like peptides, and several downstream targets of the Jun-kinase pathway involved in the oxidative stress response [114]. However, the effect of resveratrol on aging remains controversial since other studies showed that resveratrol supplementation was not able to extend mean, median, or maximum lifespan of male and female flies; also, the body composition of the flies remained largely unchanged, flies did not exhibit any improved stress response towards H_2_O_2_ exposure and the mRNA levels of antioxidant and longevity-related genes, including *Sir2*, spargel (*srl/PGC-1*), and I’m not dead yet (*indy*) remained unchanged [148].

On the other hand, it was shown that dietary ursolic acid supplementation (a triterpenoid exhibiting potential anti-inflammatory, antimicrobial, and anti-obesity properties) significantly elongated the healthspan, lifespan, and climbing activity of male *Drosophila*, probably because it counteracts age-related deficits in muscle strength. The authors also showed upregulation of the *srl/PGC-1* expression levels that triggered a metabolic shift without reducing fecundity or gut integrity. In addition, ursolic acid was also shown to affect the flies’ microbiota which resulted in lifespan extension [149].

Many other compounds have also been found to improve aged phenotypes and healthspan in the fly. Specifically, alkylresorcinols (belonging to the family of phenolic lipids), along with prunetin (a dietary isoflavone with phytoestrogenic properties), extended the lifespan of *Drosophila* and improved climbing activity [150, 151]. Prunetin-fed males exhibited increased expression of *Sir2* by 22%, AMPKα activation by 51% and elevated triglyceride levels by 29%, whereas glucose levels were decreased by 36%. As female flies are considered long-lived compared to males and exhibit higher triglyceride levels, it was thought that prunetin “feminizes” male flies via its estrogenic effects and therefore prolongs lifespan [151]. Finally, epigallocatechin-3-gallate (EGCG) derived from a green tea extract improved fitness and lifespan, as well as glucose metabolism and energy homeostasis in *Drosophila*; this green tea extract increased the mean and maximum (~ 50%) lifespan accompanied by improved fitness. These effects were followed by increased expression of *srl/PGC-1*, decreased glucose concentration, and inhibition of α-amylase and of α-glucosidase activity. Furthermore, EGCG was found to suppress the expression of *Ilp5*, phosphoenolpyruvate carboxykinase (*Pepck*), and *upd2* genes which represent major regulators of glucose metabolism and systemic energy homeostasis [152].

### Fungal and marine extracts and compounds

*Ganoderma lucidum*, *Lentinula edodes*, *Agaricus blazei*, and *Auricularia auricula-judae* are edible fungi that are used as traditional medicines in China and Philippines, as it is assumed that they have anti-aging properties and they also regulate the immune system to inhibit tumor cell growth [153, 154]. Supplementation of culture medium with 5 mg/ml extracts from *L. edodes* and *A. blazei* prolongs the lifespan of male and female flies by 6.03% and 2.69% respectively [153], while under heat stress and starvation an *A. auricula-judae* extract increased only the lifespan of female flies [154]. On the other hand, *A. auricula* extracts prolonged the lifespan of both genders, i.e., of male flies by 31.41% at 5 mg/ml and female flies by 16.85% at 20 mg/ml [153]. Finally, the extracts from *G. lucidum* extended the lifespan of male flies by 42.32% and of female flies by 29.24% at 80 mg/ml and 5 mg/ml, respectively. The dose and sex-dependent effects of edible mushroom extracts in promoting the longevity of *Drosophila* may be partially attributed to their ability to enhance antioxidant stress responses by modifying nutrient signaling pathways.

Indirubins are a family of bis-indoles naturally occurring in edible gastropod mollusks and plants, most of which are dual inhibitors of both cyclin-dependent kinases and glycogen synthase kinase-3 (GSK3; known in *Drosophila* as shaggy, *sgg*). GSK3/sgg regulates several cell functions, including survival, differentiation, proliferation, and metabolism. Accordingly, GSK3 has been implicated in various pathologies, including carcinogenesis, neurodegeneration, and diabetes. Our in vivo study of the hemisynthetic cell-permeable indirubin derivative 6-bromoindirubin-3′-oxime (6BIO) showed that 6BIO increases flies’ healthspan by modulating bioenergetic pathways and activating cytoprotective modules [155]. Our results were further validated in human cells lines, suggesting a conserved action of 6BIO mechanisms [156].

### Dietary supplements intervention in age-related diseases

*Drosophila melanogaster* has been a valuable tool to unlock mechanisms underlying the onset and progression of many age-related diseases such as cancer, diabetes, neurodegenerative disorders, kidney, and immunological diseases [157]. The best-established screening assays have been developed and performed in *Drosophila* disease models with obese or neurodegenerative phenotypes [158–160], while considering the recent discovery and characterization of oncogenes and tumor suppressor genes in the fruit fly, there is a growing interest in screening tests to identify molecules with tumor growth inhibiting properties [157, 161].

As mentioned above DR, CRMs, healthy diets (such as the Mediterranean diet), and a healthy lifestyle have been proposed to promote energy balance and reduce the risk for cardiovascular disease and diabetes. Studies in *Drosophila* have revealed several extracts and pure compounds that could reduce fat accumulation and ectopic fat distribution associated with several pathological conditions. For example, *Ilex paraguariensis* extract was suggested to reduce the detrimental effects of HFDs in *Drosophila* [162], while the extract’s metasaponins, phenolic compounds, and methylxanthines increased mean lifespan and reduced fat accumulation along with cholesterol levels [162]. Additionally, supplementation of HFDs with 4% nectarine increased lifespan and fecundity in female wild-type flies while it decreased the expression of several metabolic genes including the foxo transcriptional target *Pepck* and oxidative stress-related genes (e.g., peroxiredoxin). Moreover, nectarine extract improved the survival rates of female *Sod1* mutant flies and reduced the levels of oxidative damage [163]. Supplementation of flies HFD with 3 mg/ml of rosemary extract elevated the enzymatic activities of Sod and Cat, increased the expression of *cncC/Nrf2*; and reduced DNA lesions and MDA levels [164]. Furthermore, a recent study conducted in our lab revealed in the fly model the health beneficial properties of extra virgin olive oil (EVOO; a major component of the Mediterranean diet) on the pathological aspects of aberrant IIS activation [165], which results in increased accumulation of triglycerides in the flies’ fat body; in significant inflammatory responses and reduced longevity [166]. Oleocanthal, a compound of EVOO, showed anti-inflammatory activity in mammalian cells [167]. In support by using a *Drosophila* model, which ubiquitously overexpresses the *InR* gene, we administrated 10 μg/ml oleocanthal (a compound isolated from EVOO) in the transgenic flies’ medium, which in turn extended lifespan by reducing the toxic effects of IIS overactivation [165]. Moreover, oleocanthal exerts neuroprotective properties, and it has been suggested as a novel therapeutic strategy in neurodegeneration [168]. In addition, moderate supplementation of the flies’ medium with cocoa increases flies average lifespan under normoxia, whereas under hyperoxia or in a Cu/Zn-Sod-deficient background, cocoa exhibits a strong antioxidant activity, significantly increasing lifespan [169].

Regarding neurodegeneration, several experimental fly models have been employed to test molecules that could potentially protect against neurotoxicity or delay the progressive loss of neuronal function. These disease-mimicking models have been constructed either by genetically manipulating the *Drosophila* genome to insert mutations or human disease-causing genes or by pharmacological induction of neurodegenerative diseases [170, 171]. Specifically, deficiency of the protein deacetylase 1 (*DJ-1*, *α*, or *β*), mutation of the leucine-rich repeat kinase 2 (*Lrrk*, also known as *LRRK2*) or expression of the human synuclein alpha (*SNCA* or *h-aS*) in *Drosophila* leads in phenotypes that phenocopy Parkinson’s disease (PD) pathology [172, 173]. Moreover, chronic exposure to paraquat has been recognized as an accelerator of PD manifestation along with lifespan and neuromuscular activity suppression [137]. Accordingly, several genetic manipulations in *Drosophila*, such as the expression of constructs encoding the human amyloid beta precursor protein (*APP*) and human beta secretase 1 (*BACE1*) or the overexpression of the human microtubule-associated protein tau (*MAPT*) in the retina, have led to the generation of transgene models that imitate different aspects of the Alzheimer’s disease (AD) pathology [174]. Moreover, mutations in RNA-binding proteins of *Drosophila*, such as in the transactive response DNA-binding protein-43 (*TBPH*, also known as *TDP-43*) resemble the onset of the neurodegenerative amyotrophic lateral sclerosis (ALS).

Based on the *DJ-1-*deficient model of PD, Sanz and colleagues [175] presented recently a screening study of a wide range of small molecules, which are either known to exert health beneficial properties or used to cure other conditions, to identify therapeutic candidates for PD. Compounds were mainly tested for their ability to improve PD’s neuromuscular defects by measuring flies climbing activity [175, 176]. This study suggested that supplementation of *DJ-1-*deficient flies’ medium with dexrazoxane (6.2 μM), pterostilbene (78 μM), sodium phenylbutyrate (0.54 mM), tocopherol (1 mM), dalfampridine (1 mM), methylene blue (6 μM), or minocycline (200 μM) resulted in improvement of the distinct mobility impairment of the PD phenotype. Moreover, most of the compounds mentioned above were found to diminish cytotoxicity of *DJ-1-*deficient human neuroblastoma cells [175]. Accordingly, Casani and colleagues [177] used the same PD fly model to test several vitamins. Vitamins, as described above, are among the most popular nutrients known to vitally contribute in maintaining energy balance [178]. Both the use of 1 mM of a-tocopherol (a type of vitamin E) and 0.25 mg/ml ascorbic acid (vitamin C) for 14 days resulted in downregulation of stress markers and extension of lifespan, probably by boosting Cat activity [177]. Furthermore, Faust and colleagues [179] tested the properties of celastrol on *DJ-1* deficient flies. Celastrol is a triterpene known for its antioxidant properties, that is extracted from the root bark of the *Triperygium wilfordii*, a plant indigenous to southern China. The administration of 20 μg/ml celastrol for 20 days reduced the loss of dopaminergic neuron and brain’s dopamine levels. Since degeneration of dopaminergic neuron is a hallmark of PD [180], the antioxidant and anti-inflammatory properties of celastrol sound rather promising [179]. Consistent studies have been also conducted on the *Lrrk*-mutated fly model of PD. The G2019S mutation in the *Lrrk* gene increases its pro-oxidative activity and inhibits endogenous peroxidases. The supplementation of flies’ medium with 10 μM/ml of the strong kinase inhibitors piceatannol, thymoquinone, and esculetin reduced loss of dopaminergic neurons, oxidative load, and locomotor defects compared to weak kinase inhibitors, resulting in improved climbing scores and lifespan extension [181]. Moreover, supplementation of the *Lrrk*-mutated flies’ medium with 0.05–0.1 μΜ of lovastatin for 4 weeks activated the Akt1-cncC/Nrf2 axis and inhibited the activity of GSK3/sgg. Similarly, the *h-aS* transgenic fly model of PD was employed to test the neuroprotective activity of *Cantella asiatica* leaf extract [182]. The supplementation of flies’ medium for 24 h with 0.25–1.0 μl/ml of the extract reduced PD symptoms by delaying the loss of neuromuscular activity and lowering oxidative stress.

The best example of pharmacologically induced experimental fly model to study neurodegeneration is the long-term administration of paraquat that accelerates PD development. Peng and colleagues [59, 137, 183] have exploited this pharmacological model to test the properties of several extracts. Administration of 10 mg/ml of apple polyphenols extracts, 5 mg/ml of blueberry extract, or 30 mg/ml of black rice extract on the medium of flies chronically exposed to paraquat attenuated motor neuron degeneration along with early mortality. The authors attribute the beneficial activities of these extracts to their ability to interact with the expression of age-associated genes and antioxidant enzymes (see above) [59, 137, 183].

Curcumin and acacetin have been proposed to ameliorate the AD phenotype in several AD *Drosophila* models. More specifically, 0.01% *w*/*w* of curcumin supplementation resulted in increased healthspan and longevity of flies, while it reduced neurotoxicity by promoting amyloid fibril conversion and reduction of amyloid beta oligomeric species [184]. On the other hand, acacetin was proposed to rescue AD transgenic flies from developing motor abnormalities and decreased the number of amyloid plaques by inhibiting APP synthesis and decreasing BACE-1 activity [185].

Finally, the combined use of *Mucuna pruriens* (0.1% *w*/*w*) and *Withania somnifera* (0.1% *w*/*w*) extracts in the medium of *TBPH*-mutated flies rescued the irregular locomotion and sleep deregulation. As proposed by Maccioni and colleagues [186], results hint towards a possible deregulation of some potassium channels in the *TBPH*-mutated model of ALS that might shed new light on future therapeutic strategies.

In conclusion, these studies support the notion that supplementation of flies’ culture medium with specific natural products may either increase healthspan/lifespan and/or ameliorate some of the age-related diseases phenotypes. The beneficial effects of these dietary interventions are mainly attributed to the crosstalk of nutrient sensing or signaling modules with factors of the cellular stress-response pathways [132].

## Limitations of nutrition studies in *Drosophila*

*Drosophila* is a well-investigated and highly tractable model organism employed in nutrition research and nutraceuticals discovery since, as mentioned above, it shares high homology with several human metabolism and disease-related genes. Consistently, several insights of the molecular mechanisms that affect in vivo aging have been identified by studying the effects of distinct dietary habits and/or components of the fruit fly diet, which have been further translated or verified in mammals. Still, several considerations should be thoroughly taken into account before interpreting and consequently translating the results of nutraceutical studies from flies to humans.

### The composition of diets and the dosage of nutraceuticals

Several meta-analysis studies that tried to investigate the systemic effects of specific nutrients on healthspan and/or lifespan extension in the fly model found it hard to compare studies from different laboratories that use different “standard” *Drosophila* mediums, which exact content is rarely reported in published research papers [97, 187]. Given the number of existing nutrients in a diet and their cross-interactions, along with the established fact that even the dilution of a single amino acid can eventually modify longevity, the lifespan variations obtained in studies conducted by different research groups on the same model organism is not surprising [91]. A way out to this issue could be the use of synthetic (chemically defined) diets, like a holistic medium described recently [188], which will make nutrients and drugs more available to flies. However, a major disadvantage of this approach would be the cost and the relatively complex preparation. Thus, a compelling solution for consistency of nutrition studies in model organisms, including *Drosophila*, is the detailed reporting of the nutritional ingredients of complex diets.

Likewise, in the case of functional foods and nutraceuticals, caution should be taken regarding dosage, since different concentrations of the same molecule can yield entirely different outcomes due to mild stress (Fig. 4), a process known as hormesis [189]. This is particularly highlighted by genetic studies in which sustained induced overactivation of stress or nutrient sensors, such as cncC/Nrf2, Tor, or foxo, could have either pro-longevity or toxic effects, depending on the duration of the intervention and their expression levels [76, 132].

### Hurdles in interpreting results from dietary interventions in *Drosophila*

Another critical aspect of dietary interventions in the fly is the major role of the *Drosophila* olfactory and gustatory systems in regulating longevity. It has been proven that mutations in the olfactory system have the ability to alter energy balance, increase stress-resistance, and promote longevity [190]. Furthermore, it was suggested that the ability of flies to taste regulates lifespan expectancy. Specifically, either taste inputs or gustatory cues affect longevity by modifying a wide range of biological functions [191]. Notably, other studies indicate that the administration of various dietary factors in *Drosophila* medium, such as plant-derived secondary metabolites, results in reduced food intake as they significantly affect the taste of the food due to sweetness, bitterness, and/or saltiness [2]. Food intake can also be disturbed by the acidity of the medium, as the pH of the culture medium directly affects flies feeding behavior and modifies parameters, such as gut microbial growth, which ultimately impact on survival [192]. In addition, since food dilution to achieve CR results in the consumption of greater amounts of the diluted medium [193] and the current methods on food intake focus mainly on estimating digestion rather than more significant parameters, such as nutrients absorption and assimilation, more compelling methods need to be developed [194].

Moreover, diet intervals of the early developmental stages have been shown to have a significant role in longevity of the adult. Specifically, it was showed that the larval fat cells are used as energy sources in the early adulthood of *Drosophila* flies [195]; additional studies highlight the importance of the protein source (yeast) quality in the diet of larvae and its beneficial effects on physiological processes of the adult *Drosophila* life [196]. In support, recent studies emphasize an inter- or trans-generational consequence of diet [197–200], and the differential impact that nutritional manipulations may have to depend on the gender. This is supported by the major gender-dependent differences in the communication of the gut-brain axis, the function and components of the neuroendocrine system, the sensitivity to IIS pathway, as well as in nutrient demand and utilization [201–203]. Last, but not least, inconsistency in the results of dietary interventions may also arise from variances in the age of the experimental models, as older flies tend to consume less (compared to young flies) food [91, 204]. Finally, since the gut-microbiota (derived from food intake) plays a key role in energy homeostasis of the fly host, it is worth mentioning that during aging the density of gut-microbes increases, whereas the composition of the microbes changes according to food intake [92].

## Concluding remarks

Aging is a stochastic process and given that the doses of environmental stressors remain relatively stable during a given lifetime, it can be assumed that (excluding particular lifestyle habits, e.g., smoking) the biomolecules damage and the rate of aging are mainly affected by diet- and metabolism-derived stressors. Considering also that aging is the major risk factor for human diseases like metabolic syndromes, neurodegeneration, and cancer, as well as that diet is in fact the only feasible life-lasting applicable “intervention” in humans, the use of model organisms is particularly critical towards our effort to understand how different dietary habits affect genome (nutrigenomics) and/or proteome, and for the isolation of natural products with the potential to be used in the foreseeable future as a comprehensive and surely cost-effective mean to increase healthspan and/or lifespan.

Research in *Drosophila* has pioneered our efforts to understand developmental processes in higher metazoans and quite recently the fly has reappeared in the scene as a model organism for the study of molecular-cellular mechanisms that affect aging. Moreover, studies in *Drosophila* have started to elucidate critical parameters of the impact of diet or of the optimum doses of natural products (Fig. 4) on health outcome. Nonetheless, our attempt to promote advancement in nutrition science and nutrigenomics, and also to translate the research outcomes to humans, harbors several risks and unresolved issues. For instance, species-specific effects of nutritional manipulations should be carefully taken into consideration and, although *Drosophila* may be informative in new therapeutic discovery processes, it is necessary to have a well-defined hypothesis and a thorough perception of the fly’s limitations, e.g., differences in the blood-brain barrier permeability or lack of adaptive immunity [13, 92], in order to achieve meaningful outcomes.

**Fig. 5:**
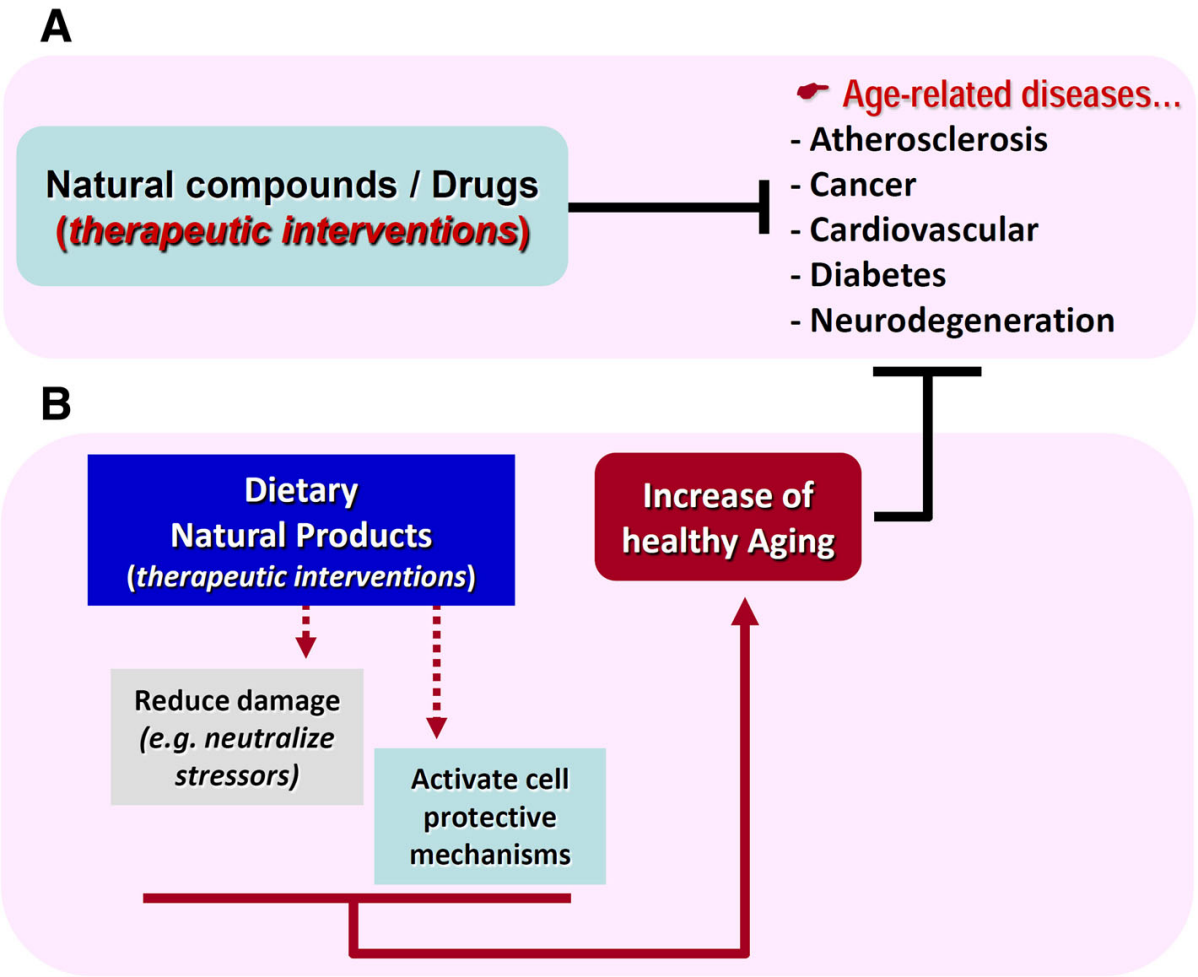
Systemic dietary anti-aging interventions have the potential to also act as inhibitors of age-related diseases. **a** Current therapeutic approaches target individual diseases that occur in an aged cellular landscape characterized by high concentration of stressors and damaged biomolecules. **b** The identification of dietary interventions, e.g., specific diets enriched in bioactive natural compounds (or extracts) that either neutralize stressors or trigger a mild activation of cytoprotective mechanisms, will likely increase healthspan suppressing thus the appearance or delaying the onset of most age-related diseases

Yet, the numerous advantages of *Drosophila* as an alternative model in nutrigenomics, as well as in modeling diet-induced chronic age-related disorders, or the effects of nutrition on aging, will surely reveal new gene-disease interactions in response to diet, and thus new targets and therapeutics. We propose that analyses of the crosstalk and functional interactions of pathways controlling genomic responses to dietary interventions in model organisms can provide valuable preclinical insights on how systemic anti-aging interventions can act as potent inhibitors of age-related diseases (Fig. 5), elucidating potential therapeutic avenues against both aging and age-associated pathologies.

## Additional file

Additional file 1: **Table S1.** List of genes. (PDF 75 kb)

## Abbreviations

6BIO: 6-Bromoindirubin-3′-oxime
AD: Alzheimer’s disease
Akh: Adipokinetic hormone
AkhR: Adipokinetic hormone receptor
ALS: Amyotrophic lateral sclerosis
AMPKα: AMP-activated protein kinase α subunit
Atg8a: Autophagy-related 8a
Cat: Catalase
cncC: Cap-‘n’-collar isoform-C
CR: Caloric restriction
CRM: Caloric restriction mimetic
DR: Dietary restriction
EGCG: Epigallocatechin-3-gallate
EVOO: Extra virgin olive oil
foxo: Forkhead box, sub-group O
GSK3: Glycogen synthase kinase-3
H_2_O_2_: Hydrogen peroxide
HFD: High-fat diet
HSD: High-sugar diet
Hsp: Heat shock protein
h-αS: Human synuclein alpha (SNCA)
IIS: Insulin/insulin-like growth factor signaling
Ilps: Insulin-like peptides
Indy: I’m not dead yet
InR: Insulin-like receptor
IPCs: Insulin-producing cells
Lrrk: Leukine-rich repeat kinase
MDA: Malondialdehyde
mth: Methuselah
NAD: Nicotinamide adenine dinucleotide
Nrf2: Nuclear factor, erythroid 2 like 2
PD: Parkinson’s disease
Pepck: Phosphoenolpyruvate carboxykinase
Rpn11: Regulatory particle non-ATPase 11
S6k: Ribosomal protein S6 kinase
sgg: Shaggy
Sirt: Sirtuin
Sod: Superoxide dismutase
srl: Spargel
TBPH: Transactive response DNA-binding protein-43 homolog
Tor: Target of rapamycin
TORC1: Target of rapamycin complex 1
TORC2: Target of rapamycin complex 2
Treh: Trehalose
upd2: Unpaired 2
