# Supplementary material for: Nutrigenomics as a tool to study the impact of diet on aging and age-related diseases: the Drosophila approach
Source: Genes Nutr. 2019 May 2;14:12. doi: 10.1186/s12263-019-0638-6 (PMC6498619; doi:10.1186/s12263-019-0638-6)
Supplement: Supplementary file 1 — Table S1. List of genes. (PDF 75 kb) [file 12263_2019_638_MOESM1_ESM.pdf]

Supplemental Table S1. List of genes

**Fly genes***			
**Name**	**Symbol**	**Also known as**	**Flybase Description**
Adipokinetic hormone	Akh		
Adipokinetic hormone receptor	AkhR		
Akt1	Akt1		AKT serine/threonine protein 1**
AMP-activated protein kinase α subunit	AMPKα	AMPK	
Autophagy-related 8a	Atg8a		
cap-n-collar isoform-C	cncC	Nrf2	
Catalase	Cat		
chico	chico		insulin receptor substrate**
DJ-1a	DJ-1a		
DJ-1b	DJ-1b		
forkhead box, sub-group O	foxo	dFOXO	
Heat shock protein 22	Hsp22		
Heat shock protein 68	Hsp68		
Heat shock protein 70	Hsp70A or Hsp70B		
I'm not dead yet	Indy		
Insulin-like peptide 1	Ilp1		
Insulin-like peptide 2	Ilp2		
Insulin-like peptide 3	Ilp3		
Insulin-like peptide 4	Ilp4		
Insulin-like peptide 5	Ilp5		
Insulin-like peptide 6	Ilp6		
Insulin-like peptide 7	Ilp7		
Insulin-like peptide 8	Ilp8		
Insulin-like receptor	InR		
lethal (2) essential for life	l(2)efl		
Leucine-rick repeat kinase	Lrrk	LRRK2	
methuselah	mth		
Phosphoenolpyruvate carboxykinase	Pepck 1 or Pepck 2[#]		phosphoenolpyruvate carboxykinase 2, mitochondrial**
Pi3K21B	Pi3K21B	PI3K	phosphatidylinositol-4,5-bisphosphate 3-kinase catalytic subunit alpha**
Regulatory particle non-ATPase 11	Rpn11		
Ribosomal protein S6 kinase	S6k	dS6K	
shaggy	sgg	GSK3	
sirtuin 1	Sirt1	Sir2	
spargel	srl	PGC-1	
Superoxide dismutase 1	Sod1		
Superoxide dismutase 2	Sod2		
Target of rapamycin	Tor		
thioredoxin peroxidase 1	Jafrac1		
Thor	Thor	4E-BP[$]	
transactive response DNA-binding protein-43 homolog	TBPH	TDP-43	
unpaired 2	upd2		

**Human genes****			
**Name**	**Symbol**	**Also known as**	
amyloid beta precursor protein	APP		
beta-secretase 1	BACE1		
synuclein alpha	SNCA	h-αS	
microtubule associated protein tau	MAPT	tau	

* flybase.org
[#] best score_ortholog in FlyBase
[$] Thor is a eukaryotic translation initiation factor 4E binding protein (flybase.org description); 4E-binding serine/threonine protein 1 (uniprot.org).
** HUGO- genenames.org
